# Interpretating SPR-Derived Reaction Kinetics via Self-Organizing Maps for Diagnostic Applications

**DOI:** 10.1021/acssensors.5c03250

**Published:** 2025-09-25

**Authors:** Jaqueline Volpe, Floriatan S. Costa, Beatriz Sachuk, Isabela Camilo, Angélica Faria, Hélida M. de Andrade, Saimon M. Silva, Dênio Souto

**Affiliations:** † Laboratório de Espectrometria, Sensores e Biossensores—Department of Chemistry, 28122 Federal University of Paraná (UFPR), Curitiba, Paraná 81530-900, Brazil; ‡ Departamento de Análises Clínicas e Toxicológicas, Faculdade de Ciências Farmacêuticas, Universidade Federal de Alfenas (UNIFAL), Alfenas, Minas Gerais 37130-001, Brazil; § Laboratório de Leishmanioses, Department of Parasitology, Institute of Biological Sciences, 74347 Federal University of Minas Gerais (UFMG), Belo Horizonte, Minas Gerais 31270-901, Brazil; ∥ Biomedical and Environmental Sensor Technology (BEST) Research Centre, La Trobe Institute for Molecular Science (LIMS), Department of Biochemistry and Chemistry, School of Agriculture, Biomedicine and Environment, 2080 La Trobe University, Melbourne, Victoria 3086, Australia

**Keywords:** surface plasmon resonance, multiepitope protein, kinetic approach, canine visceral leishmaniasis, machine learning, self-organizing map

## Abstract

Biosensors emerge as promising, cost-effective infectious disease diagnostics in resource-limited settings, requiring neither laboratory infrastructure nor specialized personnel. Surface plasmon resonance (SPR)-based biosensors remain preeminent for label-free, real-time analysis of biological interactions and kinetic parameter determination. Integrating Artificial Intelligence (AI), particularly self-organizing maps (SOMs), automates infection screening by projecting high-dimensional data onto topology-preserving 2D maps, offering advantages in diagnostic strategies by enabling efficient classification of infected vs healthy patients. This study presents an SPR biosensor with SOM analysis to enhance serodiagnosis of canine visceral leishmaniasis (CVL), a neglected tropical disease, whose delayed and inadequate detection in human and canine populations compromises effective disease control. The reaction kinetics of PQ20, a multiepitope chimeric protein with 20 B- and T-cell epitopes, with anti-PQ20 was evaluated. The proposed mechanism suggests two immunodominant epitopes of PQ20 through its reaction with polyclonal antibodies of *Leishmania chagasi*, presenting high initial association rates (*k*_a1_ = 2.4 × 10^5^ L mol^–1^ s^–1^; *k*_d1_ = 5.5 × 10^–4^ L mol^–1^ s^–1^). The biosensor’s diagnostic performance was evaluated, achieving a 5.1 nmol L^–1^ detection limit. SOM clustering indicated a higher specificity at shorter reaction times, supporting reduced diagnostic timelines (100 s) in accordance with kinetic evaluation. Finally, SOM-based data interpretation improved sensitivity and specificity compared to univariate analysis in raw serum, enhancing the assay’s ability to classify samples in more complex media, in less than 15 min analysis time. Integrating multiepitope bioreceptors with AI-driven analysis offers rapid and label-free CVL surveillance, with broader applications for the management of this infectious disease.

## Graphical abstract

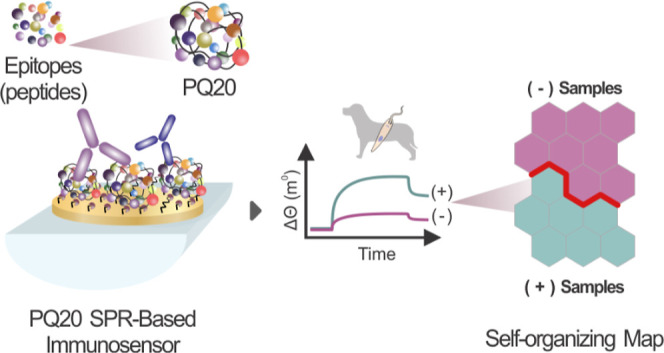

## Introduction

Considering new diagnostic tools, biosensors emerge as promising alternatives, enabling economically viable diagnostics in remote areas without requiring laboratory infrastructure or highly specialized personnel.^1–3^ Generally, biosensors are analytical devices, often portable or miniaturized, that incorporate biocomponents for identifying targets of interest through a transducing technique.^4^ Surface plasmon resonance (SPR) is an optical transducer that stands out for its real-time monitoring, label-free detection, rapid response, and straightforward sample handling capabilities.^5^ This optical technique allows detection by sensibly observing changes in the refractive index near a dielectric–metal interface through a physical phenomenon of the same name, SPR.^6^ To this day, it remains one of the leading techniques for studying biological interactions (e.g., protein–protein interactions) and determining biomolecular kinetic and thermodynamic parameters.^7,8^ Applied for medical diagnosis, the versatility of SPR-based biosensors is promising for the development of new diagnostic tools. However, to achieve its full diagnostics potential, SPR faces some limitations that need to be overcome, such as high production cost, equipment size, and limited sensitivity and selectivity of biorecognition elements, which have been quite well acknowledged and explored in the literature to improve the applicability of this class of biosensors.^9–13^

One major perspective for biosensors in infectious disease screening is integrating artificial intelligence (AI) to automate signal discrimination for improved classification of healthy versus infected patients.^14^ By exploring multivariate biosensor data through machine learning, for instance, data interpretation can be enhanced by extracting additional relevant features from the whole experimental data, thereby improving prediction accuracy.^15,16^ The self-organizing map (SOM) is an artificial intelligence technique based on unsupervised learning.^17^ It enables the projection of high-dimensional data onto a two-dimensional map.^18^ This facilitates visualization, pattern recognition, or clustering. SOM offers advantages in biomedical applications by allowing the analysis of large data sets while maintaining their topological characteristics.^19^ Additionally, it provides a simplified means of visualization and enables the automation of processes, both essential aspects for diagnostic strategies.^16^ Exploring the SOM-based artificial intelligence tool is a promising approach to enhancing SPR biosensor selectivity, especially when applied in complex media (e.g., serum, blood, or plasma).

An effective SPR signal variation is generally extracted from the sensorgrams by comparing the measured signal to a preassociation baseline, resulting in a univariate response.^20^ Although this approach is practical, it may overlook the kinetic aspects of the sensorgram. These kinetic features, reflected in the association and dissociation phases, often contain important information about the analyzed sample.^21^ The classification of the SPR data set through SOM could improve data mining and enhance diagnosis by better identifying behavior patterns in sensorgrams, exploring an intuitive 2D graph that approximates similar responses, which should differentiate negative from positive cases of a specific disease. However, to date, AI-driven analyses are still not highly exploited in SPR-based biosensors, especially SOM, which has not yet been explored yet.

This work evaluates the potential of SOM to discriminate between positive and negative cases of canine visceral leishmaniasis (CVL), a critically neglected tropical disease. The early identification or detection of asymptomatic cases of CVL canine and human visceral leishmaniasis (VL) remains a significant challenge in public health,^22^ and current diagnostic strategies have increasingly focused on exploring novel recognition elements to improve accuracy and efficiency.^23–26^

Engineered proteins and peptides, such as multiepitope proteins, have emerged as promising approaches.^27^ These proteins, also called chimeric proteins, enable the sensitive detection of specific antibodies while maintaining high selectivity, thereby minimizing cross-reactivity with other diseases. By combining multiple immunodominant epitopes, carefully selected through bioinformatics tools, multiepitope proteins offer a robust alternative to traditional diagnostic methods.^27–30^ Hence, we evaluated the application of a chimeric protein (named PQ20) as a biorecognition element for developing SPR-based biosensors for detecting antibodies against*Leishmania chagasi* (etiological agent of CVL) in canine serum. PQ20 mapped B- and T-cell epitopes of immunodominant proteins from *L. chagasi*, and it was constructed by incorporating the 20 most reactive peptides identified by enzyme-linked immunosorbent assay (ELISA) toward canine serum samples from the positive and negative groups for CVL.^31,32^ Compared to other serological assays that explore crude antigens, PQ20 detected *Leishmania* infection earlier, which could be an advancement toward a simplified diagnostic test.^33^ However, PQ20 has not yet been applied in biosensors.

Gomes and collaborators also explored the application of machine learning techniques for classifying and analyzing SPR sensorgrams through the k-NN algorithm, applied to leishmaniasis diagnosis. Their approach involved identifying key response regions, standardizing data, and enhancing the distinction between positive and negative SPR-based responses for leishmaniasis detection.^34^ However, when applied to real samples, only one positive and one negative were tested, which can be challenging when extrapolated to a broader population. Training the machine learning methods in biological samples is fundamental to avoid overfitting when expanding the model to other samples.^14^

In brief, this study builds on evaluating PQ20 to improve the accuracy and efficiency of CVL diagnosis via SPR biosensors. The kinetic behavior of this new recognition element was studied in detail via SPR, and a multivariate response analysis exploring the self-organizing map artificial intelligence tool was applied for the first time in plasmonic immunosensing. By combining engineered proteins with AI-based data analysis, we aim to enhance the predictive capability of SPR biosensors, enabling faster diagnosis and opening pathways for device automation.

## Experimental Section

### Reagents, Chemicals, and Samples

The solutions were prepared by using purified water from a Milli-Q system (ρ = 18.2 MΩ cm). 3-Mercaptopropionic acid (MPA), 11-mercaptoundecanoic acid (MUA), *N*-hydroxysuccinimide (NHS), *N*-(3-(dimethylamino)­propyl)-*N*′-ethylcarbodiimide (EDC), 2-aminoacetic acid (GLY), ethanolamine (EA), bovine serum albumin (BSA, heat shock fraction V, pH 7.0, 98%), sodium dodecyl sulfate (SDS), and phosphate-buffered saline (PBS) tablets were obtained from Sigma-Aldrich (St. Louis, MO, USA). Potassium chloride (KCl), potassium ferricyanide (K_3_[Fe­(CN)_6_]), sulfuric acid (H_2_SO_4_), sodium hydroxide (NaOH), 30% hydrogen peroxide (H_2_O_2_), acetone, isopropanol, and ethanol were purchased from Synth (Diadema, SP, Brazil).

The Leishmaniasis Laboratory kindly provided biological materials at the Institute of Biological Sciences, Federal University of Minas Gerais (UFMG). The chimeric protein (PQ20) consists of 20 peptides containing approximately 95% B-cell epitopes, as mapped using the BCPreds and ABCPred programs. The specific purified antibodies (anti-PQ20) used for analytical evaluation and biosensor construction characterization were produced by immunizing rabbits with PQ20.^31^ The composition of PQ20 consists of the following sequence of amino acids:

MWSS­QSPK­SFGSG­SGFMR­DPVRI­LGSGS­GWSR­KLGVS­FGSGS­GRMMG­VLFDY­GSGSG­FTLDG­VKYYG­SGSGF­VQKVM­MPLGS­GSGGT­EPKIK­WIGSG­SGITN­PQSTF­YGSGS­GGLID­GRYVF­GSGSG­LTYVN­GERYG­SGSGK­TKSIA­RAYGS­GSGLT­CCSLL­SYGSG­SGWLQ­QAVRY­FGSGS­GQSGQ­FRLGY­GSGSG­MRRFA­SRALG­SGSGF­TLTID­VNYGS­GSGWV­MPAYA­YLGSG­SGWLQ­QAVRY­FGSGS­GVLIE­TLKAL­GSGSG­KTGKL­LGSYG­SGSG­ISGM­GGAIY­HHH­HHH, where GSGSGs are flexible linkers in between peptides to ensure protein conformation, and HHH­HHH is a histidine tag to facilitate purification.

In total, 26 canine sera were utilized in this work, 14 collected from naturally infected cases and 12 healthy dogs, all from Belo Horizonte (MG, Brazil), confirmed by parasitological and serological tests (indirect fluorescent antibody test and ELISA), with titer determined in IFAT varied from 1:320 to 1:1280. The experimental procedures involving dogs followed animal practice by the Internal Ethics Committee in Animal Experimentation of the UFMG (CEUA Protocol n° 198/2014).

### Apparatus

SPR experiments were conducted at a controlled temperature (23 ± 1 °C) using Autolab Springle equipment (Eco Chemie, Netherlands). This equipment consists of a prism and a gold-coated glass disk (50 nm film), a He–Ne laser with an emission at 670 nm, and a photodiode detector. The configuration is based on the attenuated total internal reflection (Kretschmann configuration). The gold circular substrate can be used for up to seven individual measurements, depending on the area exposed at the center of the prism, as indicated in the user manual.

Electrochemical characterizations were performed by using a portable Metrohm Dropsens STAT-I-400 potentiostat in a conventional three-electrode electrochemical cell. The setup included a Ag/AgCl/KCl_(3M)_ reference electrode, a platinum wire counter electrode, and a gold working electrode with an area of 3.14 mm^2^. All measurements were conducted in a 5 mmol L^–1^ K_3_[Fe­(CN)_6_] solution prepared in PBS 1×.

### Biosensor Construction

The biosensor construction was carried out in six simple steps: (1) substrate cleaning, (2) functionalization of the metallic surface through SAM formation, (3) activation of the formed film for covalent anchoring of the recognition unit, (4) immobilization of PQ20, (5) blocking of remaining nonspecific sites, and (6) interaction of biorecognition receptors with the target CVL antibodies. Figure 1 presents a schematic representation of the platform used in this study for detecting anti-*Leishmania infantum* antibodies. SPR gold substrates were cleaned by immersion in piranha solution (1:3 mixture of H_2_O_2_ (30%) and concentrated H_2_SO_4_) for 1 min, followed by sequential sonication in acetone and isopropanol for 10 min each. In the electrochemical characterization, the gold electrode was first polished with alumina, followed by sonication in a 0.5% (w/v) SDS solution for 10 min and electrochemical acid cleaning via cyclic voltammetry (CV) in a 0.5 mol L^–1^ H_2_SO_4_ solution. CV was performed within a potential range of −0.1 to 1.5 V at a scan rate of 50 mV s^–1^ until the voltametric response stabilized. Under these conditions, gold’s oxidation and reduction potentials were 1.27 and 0.90 V, forming gold oxides and elemental gold, respectively.

**Figure 1:**
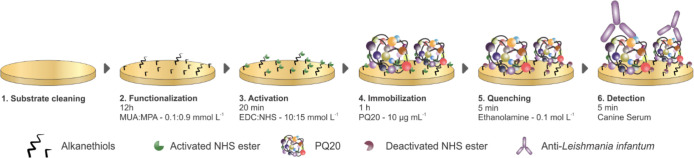
PQ20-based immunosensor construction scheme considering the (1) cleaning, (2) functionalization, (3) immobilization, (4) activation, (5) quenching, and (6) detection of CVL antibodies.

After cleaning, the gold surface of both the electrode and the SPR substrate was functionalized by forming a mixed self-assembled monolayer (SAM) by immersing the substrate in an ethanolic MPA:MUA (0.9:0.1) mmol L^–1^ solution for 12 h. The terminal carboxylic acid groups were then activated by adding an aqueous EDC:NHS solution (10:15) mmol L^–1^ for 20 min, followed by incubation with 10 μg mL^–1^ PQ20 in PBS 1× solution for 1 h to anchor the proposed bioreceptor onto the metallic surface covalently. The pH of the PBS used in the PQ20 immobilization step was evaluated across a range of 5.8 to 8.0.

A blocking step was optimized based on the ability of different agents to promote better classification between a randomly selected pool of positive and negative sera (*n* = 10 per group). The agents evaluated included 0.1 mol L^–1^ of EA at pH 8.5, 0.5% (w/v), BSA in PBS 1× at pH 7.4, and 0.1 mol L^–1^ of GLY in the same PBS buffer. It is important to note that thorough washing with ultrapure water or PBS 1× was performed between each step of biosensor assembly to ensure proper surface preparation. Once the optimized biosensor assembly was established, the prepared substrate was ready for the detection step. In this phase, after the substrate was inserted into the SPR prism, a baseline was established by adding PBS 1× (pH 7.4) until signal stabilization. Subsequently, the serum sample, in different dilutions (1:50, 1:5, and raw) prepared in PBS 1×, was introduced for 100 to 300 s to observe the association phase. Successive PBS 1× washes were then performed to remove weakly bound species, allowing the dissociation behavior to be analyzed. The sensor’s stability was evaluated by preparing the surface and storing it dry in the refrigerator (4 °C) for several days.

To verify the reproducibility of the constructed biosurface as well as the relevance of PQ20 in the identification of antibodies in clinical samples, three different substrates were evaluated according to their SPR response after the injection of both positive and negative sera at different spots of the same SPR disk, at a 1:50 dilution. In addition, two control substrates were prepared under different conditions: one by substituting PQ20 with BSA, and another in the absence of any receptor.

### SOM Analysis

Sensorgram data were acquired at a frequency of 1 Hz and were temporally aligned. To minimize potential biases associated with differences in data scale, all vectors were normalized using the min–max scaling technique, adjusting the values to the range [0, 1]. In addition to the full data set, two test intervals were considered for comparative analysis, also the association (290 to 390 s and 391 to 490 s) and dissociation phase (590 to 690 s).

The SOM was trained in batch mode using a two-dimensional grid of 32 neurons (8 × 4), following Vesanto’s approach.^35^ Quantization and topographic errors were evaluated with values below 0.46 and 0.11, respectively, indicating good data representation and adequate preservation of topology. Euclidean distance was used as the similarity metric for clustering in the two-dimensional maps, based on the spatial projection of samples onto the trained SOM. The map was generated using the SOM Toolbox (version 2.1),^36,37^ implemented in MATLAB R2023a (MathWorks, MA, USA).

### Statistical Analysis and Visualization

The graphs were processed using OriginPro software (OriginLab, MA, USA) and analyzed using a one-way ANOVA with a confidence interval of 95%. The EIS data was analyzed using ZView software (Scribner Associates, NC, USA) and modeled with an equivalent circuit. A good fit was ensured by maintaining a chi-squared (χ^2^) value below 1 × 10^–3^.

## Results and Discussion

### Characterization and Optimization of Biosensor Construction

Initially, the influence of pH on the anchoring of the PQ20 protein onto the mixed SAM composed of MUA and MPA was evaluated by using SPR (Figure 2). Based on the PQ20 sequence, its isoelectric point (pI) was estimated at 9.97. Since the monolayer-functionalized substrate contains carboxyl groups, it exhibits a negatively charged surface. Under pH conditions below its pI, PQ20 acquires a net positive charge, which may facilitate its immobilization via electrostatic interactions. Additionally, pH can significantly impact the EDC:NHS coupling reaction; therefore, three pH values were tested to assess their influence on PQ20 anchoring.^38^

**Figure 2:**
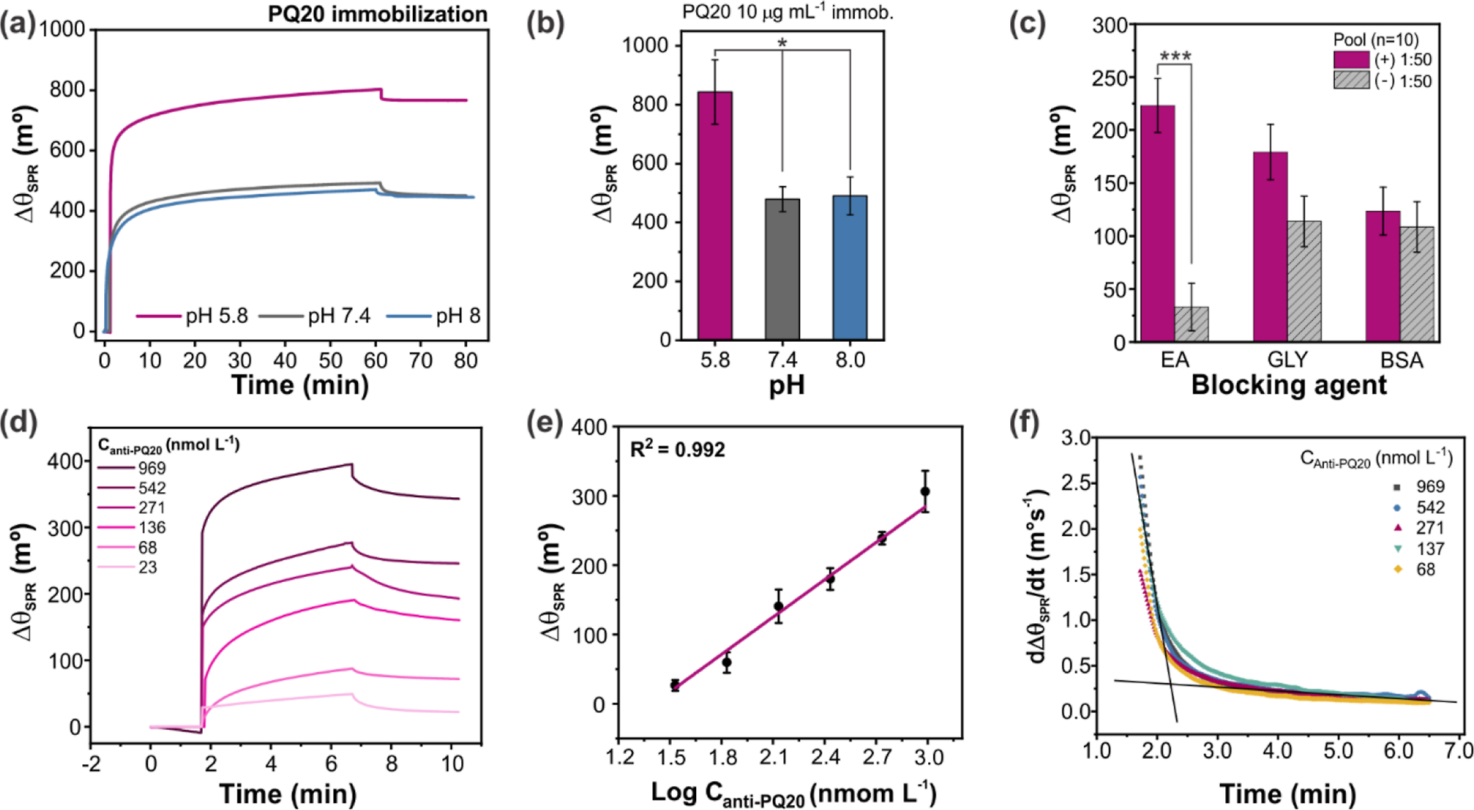
(a) Sensorgrams (Δθ_SPR_ vs Time) obtained during the immobilization of the PQ20 protein at a concentration of 10 μg mL^–1^ in PBS 1× at different pH values (5.8, 7.4, and 8.0) on an SPR sensor chip functionalized with a mixed SAM (MUA:MPA 0.1:0.9 mmol L^–1^), previously activated with EDC:NHS. (b) Practical variation values of Δθ_SPR_ according to the different pH conditions evaluated for PQ20 immobilization at the same concentration (*n*_replicates_ = 3). (c) Comparison of Δθ_SPR_ for the 1:50 dilution factors of pools (*n* = 10) of positive and negative canine samples for CVL using biosensors that employ PQ20 as a bioreceptor employing different blocking agents: GLY and EA, both at 100 mmol L^–1^, and BSA 0.5% in PBS (*n*_replicates_ = 2). (d) Sensorgrams (Δθ_SPR_ vs Time) obtained for the detection of anti-PQ20 of the proposed biosensor, through immunized rabbit serums with known concentrations of specific antibodies diluted in PBS 1×. (e) The analytical curve relating the logarithm of anti-PQ20 solution concentration versus effective Δθ_SPR_ variation (*n* = 2), presenting an angular coefficient equal to 157.2 m°, and *R* ^2^ = 0.992. (f) Variation rate with time (dΔθ_SPR_/d*t* vs time) obtained from the association phases for the different antibody concentrations illustrated in (d).

PQ20 immobilization was successful at all of the evaluated pH values (Figure 2a). The sensorgram revealed a significant increase in the SPR signal upon protein injection compared to the baseline acquired in the same PBS buffer used for protein dilution, presenting a signal-to-noise ratio of at least 300. This increase suggests that PQ20 binds to the functionalized and preactivated surface, leading to a local refractive index change. Notably, a pseudoequilibrium state was rapidly reached after protein addition, suggesting high surface coverage. After PBS washing, only a slight signal reduction was observed, indicating a strong interaction between PQ20 and the surface. However, at pH 5.8, a more pronounced increase in Δθ_SPR_ was detected (Figure 2b), suggesting a higher amount of immobilized protein at this pH, which was statistically different from the other conditions (*p* ≤ 0.039). In contrast, pH 7.4 and 8.0 exhibited similar immobilization behaviors with no significant difference (*p* = 0.998). Thus, pH 5.8 was selected as the optimal condition for PQ20 immobilization on the chosen SAM. This behavior is likely related to enhanced electrostatic interactions between the surface and the protein under more acidic conditions. At pH 5.8, the protein is expected to carry a more positive net charge, once it is under its pI, which promotes attraction to the negatively charged SAM-modified gold surface. Additionally, protein amine groups remain sufficiently protonated, while NHS-esters formed by EDC:NHS coupling are more stable at this pH, favoring the covalent reaction.

The deactivation of the remaining active sites of SAM was optimized to enhance specificity and minimize interactions with potential interferents in the sample (Figure 2c). This step was evaluated by comparing a set of samples (pool) of infected (*n* = 10) against a pool (*n* = 10) of healthy serum dog samples. Among the tested agents, EA proved to be the most effective in inhibiting residual functional groups on the activated SAM, suppressing negative responses without significantly compromising the positive signal. This indicates its efficiency in deactivating the remaining active sites, with a significant difference observed between positive and negative pool responses (*p* = 0.0002). In contrast, both glycine and BSA resulted in higher nonspecific responses with negative sera and reduced signal intensity for positive samples, showing no significant differences between the analyzed groups (*p* = 0.1325 for GLY and *p* = 0.9867 for BSA). These findings suggest that EA is the most suitable agent for this purpose, as it enhances surface selectivity, reduces nonspecific adsorption, and improves data reliability and biosensor sensitivity.

Once the optimal conditions for biosensor assembly were established, the construction steps of the proposed platform were electrochemically characterized using CV and EIS (Figure S1 and Table S1, discussion added in Supporting Information).^39–41^

The analytical response of the PQ20-based biosensor was evaluated at different concentrations of anti-PQ20 antibodies (Figure 2d) spiked into PBS. Upon introduction of the solution containing anti-PQ20, a proportional variation in Δθ_SPR_ was observed, indicating that in more concentrated solutions, a greater number of molecules interacted with the receptor, effectively altering the local refractive index. In the third phase of the sensorgram, the SPR signal decreased due to the dissociation of weakly bound species by introducing PBS, which was more prominent in higher concentrations due to the saturation of active receptor sites. Plotting the logarithm of the anti-PQ20 concentration against Δθ_SPR_ (Figure 2e) yielded a sufficient linear correlation (*R* ^2^ = 0.980). Additionally, the calculated limit of detection (LOD) and limit of quantification (LOQ) were LOD = 5.1 nmol L^–1^ and LOQ = 15.3 nmol L^–1^.

### Kinetics Approach: Proposed Mechanism for the Reaction between the PQ20 Multiepitope Chimera and *L. chagasi*-Specific Antibodies

The kinetic behavior of the reaction between the PQ20 antigen (multiepitope protein) and its specific immunoglobulins G—IgGs—(anti-PQ20) was studied, and a reaction mechanism was proposed. The processes that helped in the elaboration of the kinetic model, which explain the observed experimental behavior shown in Figure 2d, are described. Analyzing the sensorgram (Figure 2d), it can be observed that the highest response variation (Δθ_SPR_) occurs in the initial times of the reaction. From the association phases, the profile of the response variation rate with time (dΔθ_SPR_/dt vs time) was obtained (Figure 2e). It is easily evident that two response profiles are observed and that the first 100 s (approximately 1.7 min) of the association phase (time shown from 1.7 to 3.4 min in Figure 2e) is the period with the highest influence on the reaction’s kinetics.

Since the chimeric protein investigated in this work contains multiple epitopes and the antibodies (anti-PQ20) are polyclonal, more than one PQ20 binding site is likely present. This could lead to a more complex kinetic behavior than the typical 1:1 antigen–antibody interaction stoichiometry. Accordingly, the kinetic mechanism that best fitted the observed experimental data was a two-step elementary process in which a first polyclonal antibody (anti-PQ20) binds to one epitope of the bioreceptor, followed by the binding of a second polyclonal antibody (anti-PQ20*) to a different epitope of the PQ20 antigen. These reaction steps are described in eq 1 and 2.

1 PQ20+antiPQ20⇌PQ20−antiPQ20

2 $$PQ20-antiPQ20+antiPQ20^{*}⇌antiPQ20^{*}-PQ20-antiPQ20$$

eqs 1 and 2 are representatives of the proposed first and second step of the reaction, respectively, and the equilibrium dissociation constants are represented in eqs 3 and 4.

3 $$K_{D1}=\frac{k_{d1}}{k_{a1}}=\frac{\left[PQ20\right]\left[antiP20\right]}{\left[PQ20-antiPQ20\right]}$$

4 $$K_{D2}=\frac{k_{d2}}{k_{a2}}=\frac{\left[PQ20-antiPQ20\right]\left[antiPQ20^{*}\right]}{\left[antiPQ20^{*}-PQ20-antiPQ20\right]}$$

*K*_D1_ and *K*_D2_: equilibrium dissociation constant of the first and second reaction step, respectively; *k*_d1_ and *k*_d2_: kinetic dissociation constant of each respective step; *k*_a1_ and *k*_a2_: kinetic association constant. [PQ20]: concentration of the PQ20 antigen; [antiPQ20]: concentration of the anti-PQ20 antibody; [PQ20 – antiPQ20]: concentration of the conjugate PQ20 antigen – anti-PQ20 antibody, with the antibody binding to the first epitope of the antigen; [antiPQ20* – PQ20 – antiPQ20]: concentration of the conjugate antigen–antibody with the antibody binding to the second epitope of the antigen. Considering that the molecules of the PQ20 antigen are strongly immobilized on SAM/Au, its variation does not need to be considered. Upon addition of a certain amount of anti-PQ20 (antibodies) on the surface of the sensor, the formation of the conjugates antigen–antibody (PQ20 – antiPQ20 and antiPQ20* – PQ20 – antiPQ20) occurs. From this instant on, the initial concentration of the antibody (anti-PQ20) decreases according to the following equation (eq 5):

5 $$\frac{d\left[antiPQ20\right]}{dt}=-k_{a1}\left[PQ20\right]\left[antiPQ20\right]+k_{d1}\left[PQ20-antiPQ20\right]-k_{a2}\left[PQ20-antiPQ20\right]\left[antiPQ20^{*}\right]+k_{d2}\left[antiPQ20^{*}-PQ20-antiPQ20\right]$$

In a similar manner, the formation of the conjugate’s antigen–antibody (PQ20 – antiPQ20 and antiPQ20* – PQ20 – antiPQ20) can be represented as eqs 6 and 7:

6 $$\frac{d\left[PQ20-antiPQ20\right]}{dt}=k_{a1}\left[PQ20\right]\left[antiPQ20\right]-k_{d1}\left[PQ20-antiPQ20\right]-k_{a2}\left[PQ20-antiPQ20\right]\left[antiPQ20^{*}\right]+k_{d2}\left[antiPQ20^{*}-PQ20-antiPQ20\right]$$

7 $$\frac{d\left[antiPQ20^{*}-PQ20-antiPQ20\right]}{dt}=k_{a2}\left[PQ20-antiPQ20\right]\left[antiPQ20^{*}\right]-k_{d2}\left[antiPQ20^{*}-PQ20-antiPQ20\right]$$

Since the same PBS buffer solution at pH 7.4 was used for both the dissolution of the biological sample and the SPR analysis, the variations of the angle of resonance (Δθ_SPR_) can be attributed solely to the interactions that occurred on the sensor’s surface. Taking into account that only the conjugates PQ20 – antiPQ20 and antiPQ20* – PQ20 – antiPQ20 alter the Δθ_SPR_, the values observed experimentally (Figure 2d) can be attributed by the individual contribution of each interaction, Δθ_SPR1_ and Δθ_SPR2_ (eq 8), respectively, represented by eqs 9 and 10:

8 $${\Delta \theta }_{S\mathrm{PR}}={\Delta \theta }_{SPR1}+{\Delta \theta }_{SPR2}$$

9 $${\Delta \theta }_{SPR1}\propto \left[PQ20-antiPQ20\right]$$

10 $${\Delta \theta }_{SPR2}\propto \left[antiPQ20^{*}-PQ20-antiPQ20\right]$$

Being the Δθ_SPR_ rate also described by the individual contributions following eqs 11–13:

11 $$\frac{{d\Delta \theta }_{SPR}}{dt}=\frac{{d\Delta \theta }_{SPR1}}{dt}+\frac{d{\Delta \theta }_{SPR2}}{dt}$$

12 $$\frac{{d\Delta \theta }_{SPR1}}{dt}\propto \frac{d\left[PQ20-antiPQ20\right]}{dt}$$

13 $$\frac{d{\Delta \theta }_{SPR2}}{dt}\propto \frac{d\left[antiPQ20^{*}-PQ20-antiPQ20\right]}{dt}$$

Since Δθ_SPRmax_ ∝ [PQ20]_0_, performing the substitutions in eqs 6 and 7, and through the adequate rearrangement, it was possible to reach the differential equations (eqs 14 and 15), which represent the contribution of the PQ20 – antiPQ20 and antiPQ20* – PQ20 – antiPQ20 interaction, respectively.

14 $$\frac{{d\Delta \theta }_{SPR1}}{dt}=\alpha_{1}{{\Delta \theta }_{SPR1}}^{2}+\beta_{1}{\Delta \theta }_{SPR1}+\gamma_{1}$$

15 $$\alpha_{1}=k_{a2};\beta_{1}={-k}_{a1}C-k_{d1}k_{a2}{\Delta \theta }_{SPRmax}{+k}_{a2}{\Delta \theta }_{SPR2};\gamma_{1}=k_{a1}C{\Delta \theta }_{SPRmax}-k_{a1}C{\Delta \theta }_{SPR2}+k_{d2}{\Delta \theta }_{SPR2}\frac{d{\Delta \theta }_{SPR2}}{dt}=\alpha_{2}{\Delta \theta }_{SPR2}+\beta_{2}$$

$$\alpha_{2}=-k_{a2}{\Delta \theta }_{SPR1}-k_{d2};\beta_{2}=k_{a2}{\Delta \theta }_{SPR1}{\Delta \theta }_{SPRmax}k_{a2}{{\Delta \theta }_{SPR1}}^{2}$$

According to eqs 14 and 15, it was possible to verify that the contribution of the formation of PQ20 – antiPQ20 (eq 14) exhibits a quadratic behavior, while the contribution of the formation of antPQ20* – PQ20 – antiPQ20 (eq 15) exhibits a linear behavior.

Figure S2 shows the profile of the response variation rate with response variation (dΔθ_SPR_/d*t* vs Δθ_SPR_) obtained for the first 100 s (∼1.7 min) of the association phase, which is the period with the greatest contribution on the reaction’s kinetics. It is possible to observe that the same quadratic profile is observed for different antibody concentrations. From Figure S2, each curve corresponding to a given antibody concentration was fitted using the developed differential equation (eq 14). By solving this equation, the values of the kinetic constants were obtained and are inserted in Table 1. By analyzing carefully these values (Table 1), it is possible to observe that they are in concordance with the mechanisms discussed about the kinetics of the reaction proposed for the interaction between the PQ20 and its specific IgGs (anti-PQ20). As demonstrated, the reaction between these biomolecules occurs in two steps: eq 1 and eq 2. The high value of *k*_a1_ (2.39 × 10^5^ L mol^–1^ s^–1^) and the low value of *k*_d1_ (5.36 × 10^–2^ s^–1^) evidence that the formation of PQ20 – antiPQ20 (eq 1) is the fast step of the reaction. The low value of *K*_D1_ (2.24 × 10^–7^) suggests that initially an anti-PQ20 (antibody) binds strongly to one of the epitopes of the PQ20 (eq 1). In turn, low values were obtained for both *k*_a2_ (5.49 × 10^–4^ L mol^–1^ s^–1^) and *k*_d2_ (5.50 × 10^–4^ s^–1^), showing that the second elementary step, which involves the formation rate of the antiPQ20* – PQ20 – antiPQ20 complex (eq 2), is much slower than the first step. Furthermore, the higher *K*_D_ value for the second step suggests that another antibody, with a much lower affinity, may have bound to a second immunodominant epitope of the multiepitope protein. From the general equation (*K*_D1_ × *K*_D2_: 2.24 × 10^–7^ mol L^–1^), it is possible to suggest high binding affinity between the PQ20 and its specific antibodies against *L. chagasi*, quantitatively proving the strong immunogenic character of this chimeric multiepitope protein and its potential use in the immunodiagnostic of CVL.

**Table 1:** Kinetic and Thermodynamic Parameters Obtained by Solving the Equation Developed from the SPR Results for the Antigen–Antibody (PQ20 and Anti-PQ20) Reaction. *k*_a1_ and *k*_a2_: Association Kinetic Constants; *k*_d1_ and *k*_d2_: Dissociation Kinetic Constants; K_D1_ and K_D2_: Equilibrium Dissociation Constants^a^

*k*_a1_ (L mol^–1^ s^–1^)	*k*_d1_ (s^–1^)	*K*_D1_ (mol L^–1^)	*k*_a2_ (L mol^–1^ s^–1^)	*k*_d2_ (s^–1^)	*K*_D2_ (mol L^–1^)
2.39 × 10^5^	5.36 × 10^–2^	2.24 × 10^–7^	5.49 × 10^–4^	5.50 × 10^–4^	0.998

a The deviations found for these measurements were not significant (<5%).

PQ20 contains several amino acids, designed by combining 20 T- and B-cell epitopes linked by flexible Gly-Ser linkers and a His-tag, with in silico predictions indicating that 95% of its residues correspond to B-cell epitopes. The flexible linkers favor epitope accessibility and may allow simultaneous binding of multiple IgG molecules, consistent with the high serological reactivity of PQ20 in ELISA.^31,33,42^ In the SPR sensor, PQ20 was covalently immobilized on a mixed SAM via amine coupling, providing additional spacing from the gold surface and reducing the steric hindrance, which supports the plausibility of multivalent binding. The kinetic analysis was simplified into a sequential two-step binding model to extract apparent rate constants while acknowledging that multiple binding sites and potential crowding effects could also contribute to the observed interaction.^43^ Next, the PQ20-based SPR biosensor was applied to detect antibodies in CVL-positive and CVL-negative canine sera.

### PQ20-Based SPR Biosensor Performance in Clinical Samples

Before conducting the analysis of individual samples, the reproducibility test demonstrated that, in all substrates evaluated, the positive response was consistently higher than the negative (*p* <0.02), with no statistically significant differences observed among the prepared disks (Figure S3). Furthermore, the control experiments, performed either in the absence of a receptor or by using BSA as the recognition element, highlighted the importance of PQ20 in discriminating positive and negative cases. Under these conditions, no statistically significant differences between positive and negative samples were observed (*p* >0.09).

Following the optimization results previously discussed (Figure 2c), the use of BSA as a blocking agent appears to introduce two potential limitations. First, it may not act as an inert blocker, as experiments in which PQ20 was replaced with BSA resulted in similarly high responses for both positive and negative sera, suggesting that the BSA layer could capture serum components nonspecifically. Second, due to its relatively large molecular size (∼66.5 kDa), BSA may cause steric hindrance on the functionalized surface, thereby reducing the accessibility of immobilized bioreceptors to the target analyte. Comparable effects have been suggested in previous studies, where bulky protein blockers were reported to hinder smaller recognition elements and compromise the biosensor performance.

Then, the optimized platform was used to evaluate individually different sera from infected (n = 14) or healthy (n = 12) dogs (Figure S4). The sample groups showed a significant expressive difference (*p* = 7 × 10^–8^), with a higher variance observed in the positive samples compared to the negative ones, a behavior expected due to the individual immune response of each dog. To discriminate the samples, an optimal cutoff value of 63.03 m° was calculated using the formula 3 × SD + *x*, where “*x*” is the mean response observed by the sensor for the negative samples, and ‘SD’ is their standard deviation. Using the cutoff value of 63.03 m° to differentiate seropositive from seronegative responses, all evaluated samples were correctly identified, resulting in 100% specificity and sensitivity. Although the absolute antibody concentration in serum was not directly determined, the fact that the SPR response at a 1:50 dilution was consistently higher for positive than for negative samples indicates significant nonspecific interactions were not occurring at the surface under this condition. This also suggests that antibodies were present within the nanomolar range at this dilution (20–500 nmol L^–1^), corresponding to approximately 0.1–25 μmol L^–1^ in undiluted sera. This estimation was derived from the calibration curve constructed with known concentrations of anti-*Leishmania* antibodies (Figure 2d), in which the SPR signals observed for the serum samples fell within the same range. The PQ20-based platform maintained stability for at least 7 days when the biofunctionalized SPR substrate was refrigerated at 4 °C between measurements (Figure S5).

Different bioreceptors have been explored in recent years to develop biosensing devices aimed at detecting antibodies for diagnosing visceral leishmaniasis, as summarized in Table S2.^23,30,32,44–53^ Most studies have focused on the use of various proteins produced through recombinant technology, as their production can be more cost-effective, safer, and customizable to meet specific requirements when compared to crude soluble antigens.^30,44–47,54^ Despite the present work, our group also explored the utilization of other multiepitope protein, called CP10, with a focus on the kinetic evaluation through SPR and QCM.^30^ In previous work, PQ10 (also called CP10) and PQ20 were explored in an ELISA immunoassay, with indications that the chimeric proteins could improve serological tests in detecting early stages of the infection.^33,55^ Thus, the current study explores for the first time the use of PQ20 for the development of a biosensor.

### New Strategy for the CVL Biosensor Using a Self-Organizing Map

Once the refractive index changes proportionally to mass changes in the sensing area, it is possible to observe that the targeted sample is added through the intense initial changes in θ_SPR_ in the association phase, corresponding to the binding rate of the biomolecules on the surface, mainly via interaction with the receptor (Figure 3a). It is important to highlight that when analyzing canine serum samples, it is crucial to account for potential matrix effects, as target antibodies exist within a complex biological environment, leading to nonspecific interactions. After the binding event, a buffer can be added to evaluate the unbinding of weakly bound species, corresponding to the dissociation phase.^56,57^

**Figure 3:**
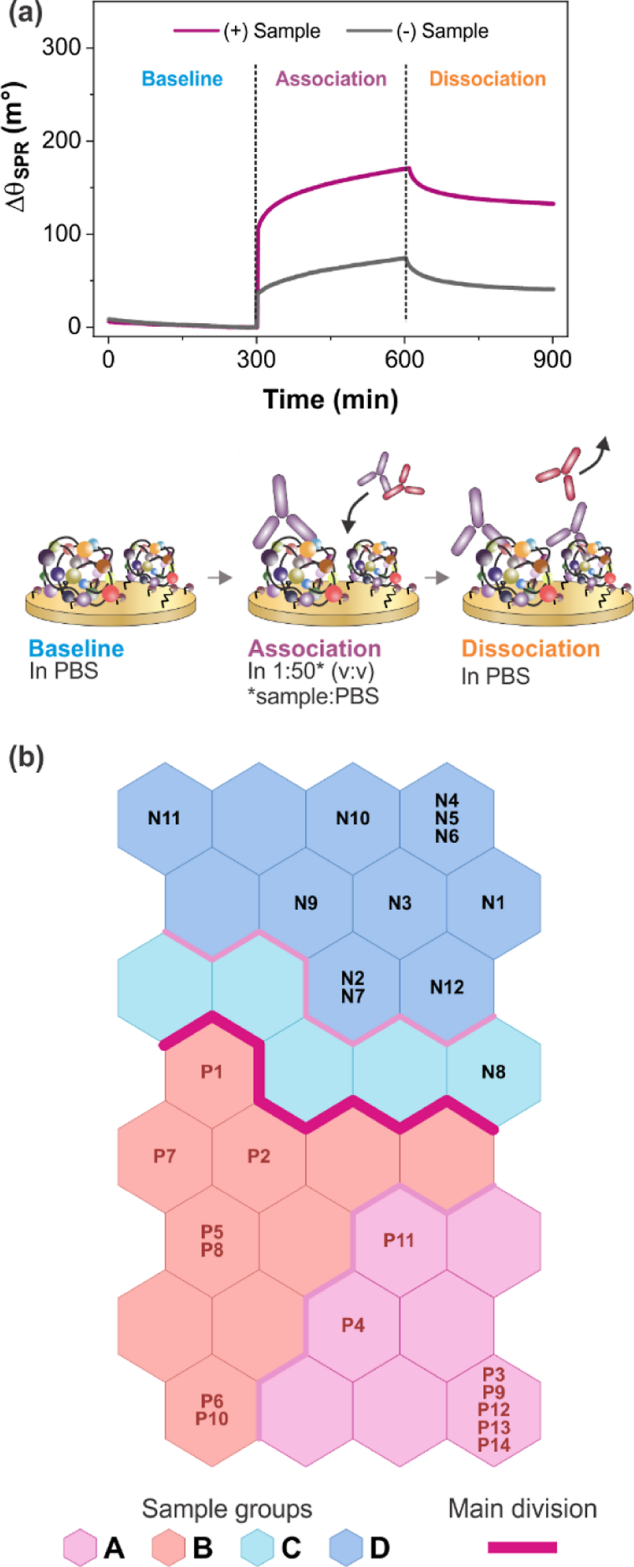
(a) Sensorgrams (Δθ_SPR_ vs Time) of the PQ20-based proposed biosensor for a positive and negative case diluted in PBS 1:50 (v:v) for CVL and the respective phases—baseline, association, and dissociation—highlighted. (b) Spatial distribution of the canine serum samples, considering the SOM grid for the association phase only (from 290 to 390 s). From P1 to P14 are the positive samples, and from N1 to N12 are the negative ones.

Generally, a univariate evaluation is performed on real-time SPR sensorgrams by considering the effective Δθ_SPR_ variation after completion of both association and dissociation events. However, this may neglect the kinetic and thermodynamic aspects of the biomolecular interaction studied once this information affects the curve behavior at the association and dissociation phases observed in sensorgrams (Figure 3a).

Hence, the evaluation of association and dissociation in biologically derived samples presents significant challenges. In this study, we propose incorporating the entire θ_SPR_ variation over time into the data analysis to enhance discrimination accuracy and assess the influence of association and dissociation stages on sample discrimination by using an SOM-based artificial intelligence tool to classify infected and healthy canine serum samples through SPR biosensor results. To better understand the contribution of each sensorgram stage to sample classification, SOM analysis was explored using sera that had previously been correctly classified by univariate analysis at a 1:50 dilution. Four different timeframes were tested: the whole sensorgram (290–700 s, Figure S6b), the association phase (290–390 s, Figure 3b), the dissociation phase (590–690 s, Figure S6c), and a partial association interval (391–490 s, Figure S6d). During the association phase, the interaction of specific antibodies with the PQ20-functionalized surface produced a marked increase in the intensity of the SPR signal, evidencing its strong influence on the refractive index variation of infected sera. In the SOMs, positive samples with greater impact on the association angle variation were consistently clustered in the lower region, while negative controls appeared in the upper region, a trend observed across all tested timeframes (Figure S6), as also represented in the component planes generated from 290 to 700 s (Figure S6a).

Interestingly, when only the first 100 s of association were used (Figure 3b), the SOM correctly classified all samples, whereas models based on the entire sensorgram or the dissociation interval resulted in misclassifications (e.g., samples P1 and P2 clustered with negatives; Figure S6b,c). These findings corroborate the kinetic analysis, which revealed a two-step binding behavior. This possibly explains why longer monitoring periods, particularly during dissociation, led to a reduced classification accuracy.

Finally, when focusing on an intermediate association window (391–490 s, Figure S6d), the classification efficiency decreased, further reinforcing the importance of early binding events for enhancing device selectivity. Overall, these results highlight how kinetic behavior directly shapes SOM-based data interpretation, emphasizing that the most informative time frame for accurate diagnosis is during the initial fast binding phase.

It is important to note that the data analysis using SOM did not necessarily yield better classification metrics compared to the previously evaluated univariate model when analyzed with 1:50 dilution. To evaluate (a) whether the posthoc analysis of sensorgram phases provided crucial information for predictive capacity by accounting for nonspecific interactions and (b) whether, in more complex samples, the SOM could outperform univariate data analysis by identifying signal relationships beyond the effective angle variation at the end of the detection event, an assay with more concentrated serum was performed using, initially, a set of seven positive and seven negative samples.

With all analyses performed on the same set of samples (Table 2), it was observed that in more complex media, using sera diluted only 5-fold or not diluted at all, the ability of the surface to differentiate positive from negative cases decreased when relying solely on Δθ_SPR_.

**Table 2:** Effect of Association Time and Dilution Factor on the Correct Classification of Positive (*n* = 7) and Negative (*n* = 7) Samples, Showing *p*-Value, ROC Area under the Curve, Cut-Off Value, Sensitivity, and Specificity^a^

time (s)	DF (v/v)	*p*	ROC	Δθ_cutoff_ (m°)	sensit. (%)	spec. (%)
300	50	<0.001	1	63	100	100
100	5	0.07	0.77	222	85.7	71.4
300	5	0.78	0.16	466	28.6	100
100	whole	0.02	0.84	333	71.4	100

a Abbreviations: DF, dilution factor; ROC, receiver operating characteristic area under the curve; Sens., sensitivity; Spec., specificity.

This outcome was expected, since in more complex media, nonspecific binding events may occur and hinder specific interactions, as reflected by a significant increase in the cutoff value required for classification. The effect of sample concentration was further evidenced by a lower ROC area under the curve, poorer sensitivity and specificity values, and a weaker statistical difference between groups, as indicated by the *p*-value when compared to the 1:50 dilution. Moreover, for the 1:5 dilution, the relationship between time and predictability was evident, as longer association times resulted in poorer signal segregation with the highest *p*-value, the lowest ROC area, and reduced sensitivity. Therefore, the association phase proved crucial for interpreting SPR data by serum analysis, with SOM demonstrating the potential for data curation in SPR-based biosensor responses. In this context, just 100 s of interaction was sufficient for accurate data interpretation. This finding is interesting for faster incubation times in future plasmonic biosensing applications, even in stationary setups such as the one used in this work. Once the association and dissociation kinetics constants of nonspecific reactions usually indicate a slower reaction, small times are essential to minimize matrix effects in biosensors applied as diagnostic tools.

Once it was observed that undiluted serum with 100 s of interaction allowed a certain level of classification, the SOM capacity to discriminate CVL cases in more complex media was further evaluated using a raw serum broader sample group. In this case, the entire sensorgram data set was considered, and the resulting classification was compared with that obtained by univariate analysis (Figure 4).

**Figure 4:**
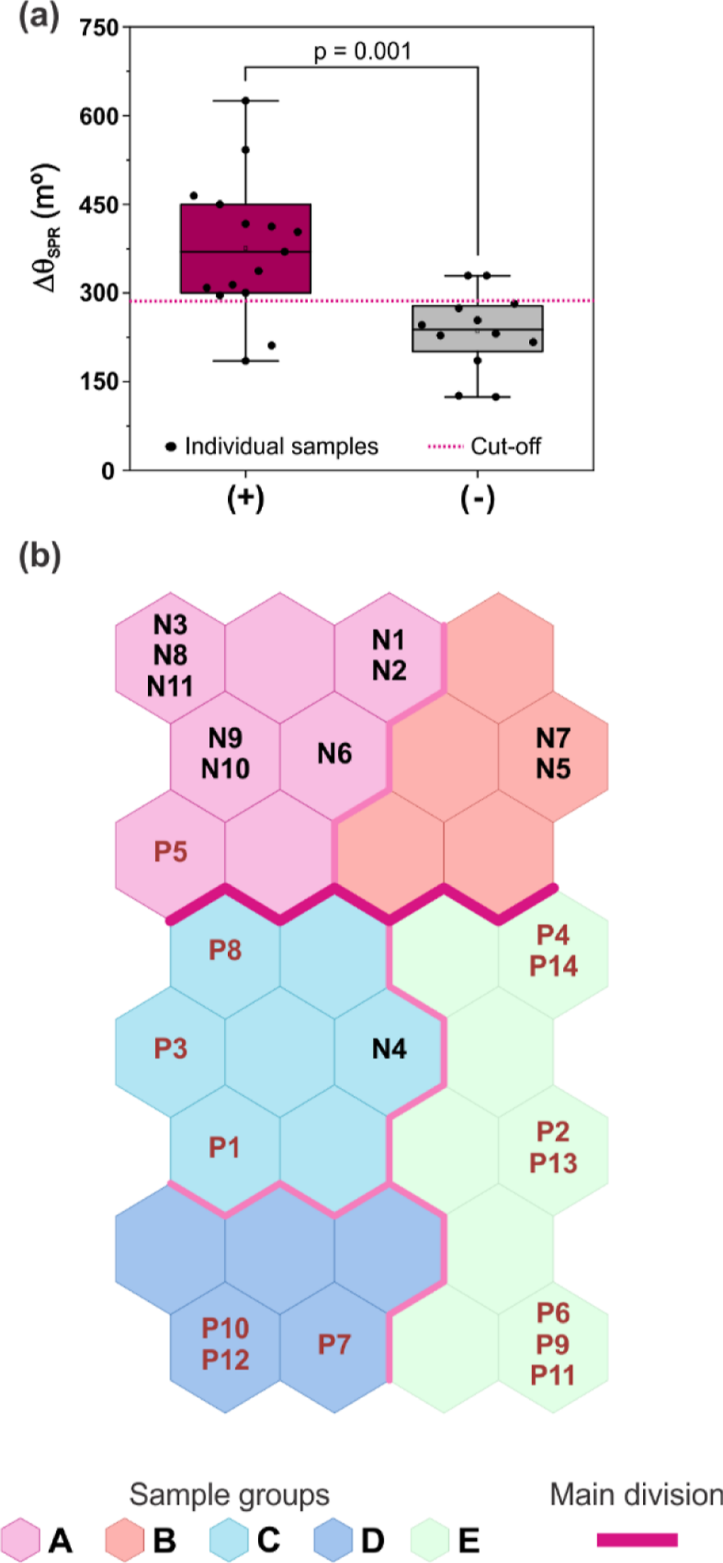
(a) Positive and negative responses observed for positive (*n* = 14) and negative (*n* = 11) samples undiluted, represented in box plots. (b) Spatial distribution of the canine serum samples, considering the SOM grid of the whole sensorgram. From P1 to P14 are the positive samples, and from N1 to N11 are the negative ones.

Neither method was able to correctly predict all of the tested samples, which is expected in more complex scenarios. For the univariate analysis (Figure 4a), a statistically significant difference between positive and negative groups was observed (*p* = 0.001). Using a cutoff of 288.0 m°, determined by the ROC curve with the highest Youden index, the analysis achieved 85.7% sensitivity and 81.8% specificity, with a total of four samples misclassified. In contrast, the SOM misclassified only two samples, yielding predicted sensitivity and specificity values of 92.8% and 90.9%, respectively (Figure 4b). The same samples were also incorrectly classified by a univariate analysis. However, when examining the effective SPR signal variation, both misclassified samples showed angle responses very similar to another case that was wrongly assigned by the classical method but correctly identified by the SOM.

This finding indicates that the effective signal was not the only aspect captured during the training. Additional information, likely linked to the behavior of the association and dissociation phases, also contributed to the improved classification. Such information is particularly valuable for interpreting SPR data in simplified or miniaturized devices. Once properly trained, the algorithm can compensate for bulk effects and nonspecific interactions, allowing accurate classification without prior signal subtraction.

## Conclusion

The integration of biosensing technologies presents a promising strategy for advancing classical diagnostic methods, particularly by enhancing the predictability in immunoassays. In this study, we demonstrated the successful application of a chimeric protein (PQ20) in a plasmonic biosensor to effectively discriminate between positive and negative cases of canine visceral leishmaniasis. Incorporating artificial intelligence through SOM for SPR data analysis introduces an unprecedented approach within this context. The results also emphasize the importance of biomolecular interaction kinetics in optimizing diagnostic performance, as association times were shown to impact the platform’s selectivity significantly. These findings underscore the potential of combining biosensing strategies with AI-driven data analysis to enable more accurate, selective, and scalable diagnostic tools. In the future, SOM-based analysis of the SPR can help to understand immunological patterns since it permits spatial clustering of similar responses, as well as help in multiplexed strategies and point-of-care analysis, which aim to identify multiple diseases in one diagnostic test.

## Supplementary Material



## Notes

Electrochemical parameters obtained for EIS data analysis (PDF)

## Abbreviations

AI: artificial intelligence
BSA: bovine serum albumin
CV: cyclic voltammetry
CVL: canine visceral leishmaniasis
EA: ethanolamine
EDC/NHS: 1-ethyl-3-(3-(dimethylamino)­propyl)­carbodiimide/*N*-hydroxysuccinimide
EIS: electrochemical impedance spectroscopy
GLY: glycine
LOD: limit of detection
LOQ: limit of quantification
MPA: 3-mercaptopropionic acid
MUA: 11-mercaptoundecanoic acid
*n*_dl_: ideality parameter of the constant phase element (CPE) associated with the double layer
NTDs: neglected tropical diseases
PBS: phosphate-buffered saline
pI: isoelectric point
*Q*_DL_: double-layer capacitance
*R*_CT_: charge transfer resistance
ROC: receiver operating characteristic
*R*_S_: total cell resistance
SAM: self-assembled monolayer
SOM: self-organizing map
SPR: surface plasmon resonance
VL: visceral leishmaniasis.
