# Supplementary material for: Interpretating SPR-Derived Reaction Kinetics via Self-Organizing Maps for Diagnostic Applications
Source: ACS Sens. 2025 Sep 25;10(10):8129–39. doi: 10.1021/acssensors.5c03250 (PMC12560123; doi:10.1021/acssensors.5c03250)
Supplement: Supplementary file 1 [file se5c03250_si_001.pdf]

Supporting information:

Interpretating SPR-Derived Reaction Kinetics *via* Self-Organizing Maps for Diagnostic Applications

Jaqueline Volpe[a], Floriatan S. Costa[a], Beatriz Sachuk[a], Isabela Camilo[a], Angélica Faria[b], Hélida M. de Andrade[c], Saimon M. Silva[d], Dênio Souto[a]*

[a] Laboratório de Espectrometria, Sensores e Biossensores - Department of Chemistry, Federal University of Paraná (UFPR), Curitiba, PR, 81530-900, Brazil

[b] Departamento de Análises Clínicas e Toxicológicas, Faculdade de Ciências Farmacêuticas, Universidade Federal de Alfenas (UNIFAL), Alfenas, MG, 37130-001, Brazil

[c] Laboratório de Leishmanioses, Department of Parasitology, Institute of Biological Sciences, Federal University of Minas Gerais (UFMG), Belo Horizonte, MG, 31270-901, Brazil

[d] Biomedical and Environmental Sensor Technology (BEST) Research Centre, La Trobe Institute for Molecular Science (LIMS), Department of Biochemistry and Chemistry, School of Agriculture, Biomedicine and Environment, La Trobe University, Melbourne, Victoria 3086, Australia

***Corresponding author:**

Tel.: +55 41 3361-3297

E-mail address: denio.souto@ufpr.br

Electrochemical analysis

Each stage of the process influenced the faradaic reactions at the electrode-solution interface following the addition of different species. The deposition of insulating molecules on the gold electrode surface hindered charge transfer of the electrochemical probe $[Fe(CN)_6]^{-3}$/$[Fe(CN)_6]^{-4}$, as evidenced by a decrease in total system current in cyclic voltammetry and a shift in oxidation and reduction potentials in each necessary step for biosensor construction (**Fig. S1a**).

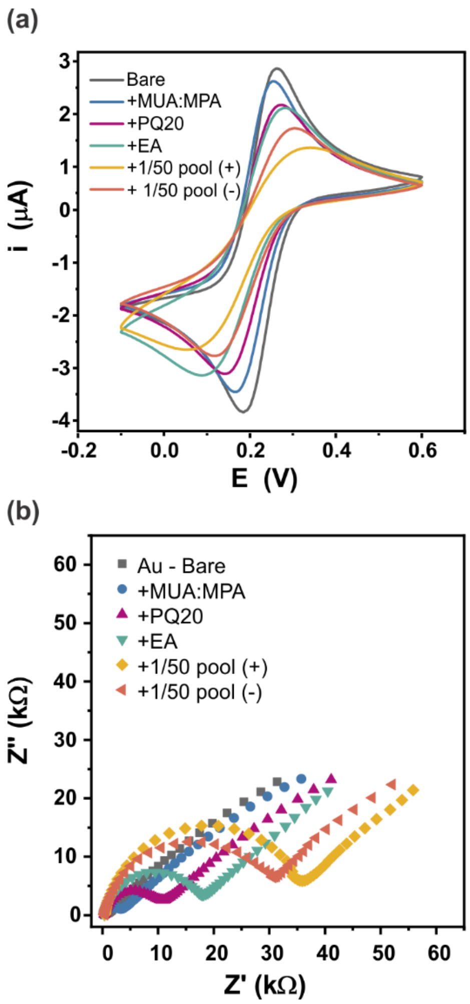

Fig. S1. (a) CVs obtained for each step of the biosensor construction for CVL, at a scan rate of 20 mV s^{-1}. **(b)** Corresponding Nyquist diagrams at the same stages, recorded at the mean potential. Both techniques were performed in a 0.1 mol L^{-1} PBS solution (pH 7.4) containing 5 mmol L^{-1} of $K_3[Fe(CN)_6]$. In the detection step, a 1/50 solution in PBS from a pool of dogs infected with CVL was used, against a pool of healthy dogs.

The largest current and potential variations were observed upon the addition of positive serum. In comparison with the negative pool, the positive samples induced a greater impact on the kinetics of the electrochemical reaction, which is likely associated with the incorporation of new molecular structures, such as specific antibodies at the electrode interface. In contrast, the negative sera did not promote such pronounced interfacial modifications, resulting in a less

significant faradaic effect. Additionally, the Nyquist diagram, obtained by EIS, revealed an increase in the semicircle, indicating higher charge transfer resistance (**Fig. S1b**). These results, particularly in the detection step of infected sera (pool), demonstrate a robust biosensor response using PQ20, reinforcing the efficiency of the platform construction process with a chimeric protein as a bioreceptor. Randles equivalent circuit analysis determined the charge transfer resistance (R_{CT}), double-layer constant phase element (Q_{DL}), and the deviation of this element from an ideal capacitor (n_{DL}) as summarized in **Tab. S1**.[39]

Tab. S1. Parameters (R_{CT}, Q_{DL}, and n_{DL}) obtained through equivalent circuit modeling using the Randles circuit from the impedance data in Fig.4b, along with their respective χ^2 values.

**Step**	**Q_{DL} / μF s^{n-1}**	**n_{DL}**	**R_{CT} / Kω**	**X^2**
(1) Au	1,01	0,88	0,474	$1,1 \times 10^{-3}$
(2) +SAM_{mix}	0,946	0,86	2,57	$1,6 \times 10^{-3}$
(3) +PQ20	0,595	0,89	9,16	$7,0 \times 10^{-4}$
(4) +EA	0,631	0,87	16,7	$7,0 \times 10^{-4}$
(5) +Positive Serum 1:50	0,584	0,89	34,5	$1,1 \times 10^{-3}$
(5) +Negative Serum 1:50	0,746	0,87	29,3	$9,1 \times 10^{-5}$

Incorporating new structures on the surface led to a slight decrease in Q_{DL} and a significant increase in R_{CT}.[40] Despite the variation in Q_{DL}, no consistent trend was observed in n_{DL}, possibly indicating that the surface morphology was not significantly altered. The observed changes in R_{CT} and Q_{DL} are expected, as molecules attached to the surface influence the electroactive interface area and hinder the electrochemical reaction at the interface.[41]

When comparing the responses of negative and positive sera by electrochemistry measurements, a greater variation was observed for the positive samples, suggesting that the surface interacts with specific antibodies related to

CVL. Nevertheless, it is important to emphasize that, when compared to SPR, the responses obtained by CV and EIS appeared less selective toward the antibody. This highlights the selectivity advantage of the optical technique for this application.

SPR analysis

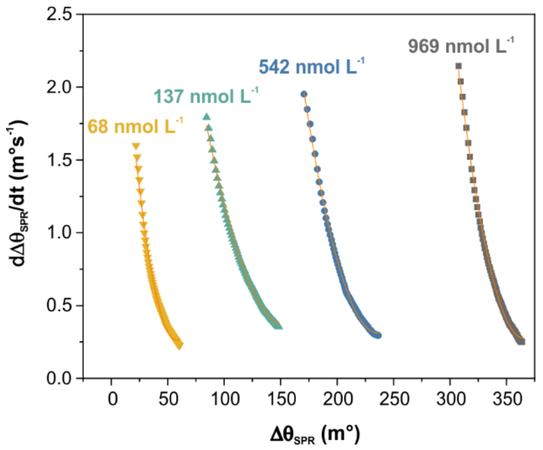

Fig. S2. Variation rate with response variation ($d\Delta\theta_{SPR}/dt$ vs. $\Delta\theta_{SPR}$) obtained for the first 100 seconds (~ 1.7 min) of the association phase for the different antibody concentrations. The fitting line represents the curves calculated from the proposed kinetic model.

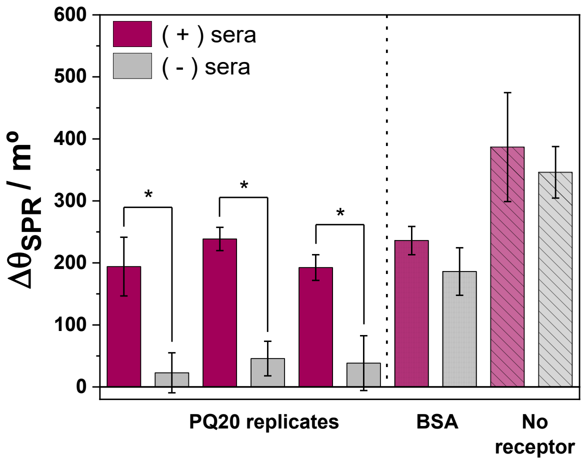

Fig. S3. Responses of positive and negative sera (n = 3) in three different substrates modified with PQ20, showing a statistically significant difference between positive and negative samples ($p < 0.05$). The respective controls correspond to the same positive and negative samples tested on surfaces prepared either in the absence of the receptor or with BSA substituting PQ20.

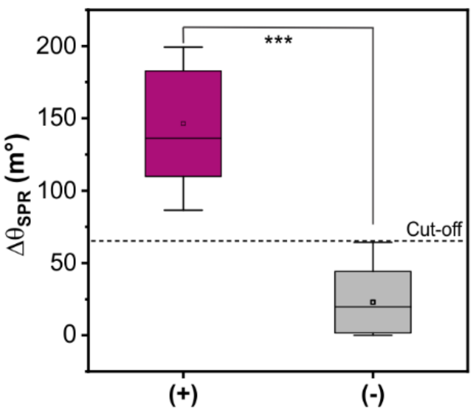

Fig. S4. Positive and negative responses observed for positive (n = 14) and negative (n = 12) samples diluted 50X, represented in box plots.

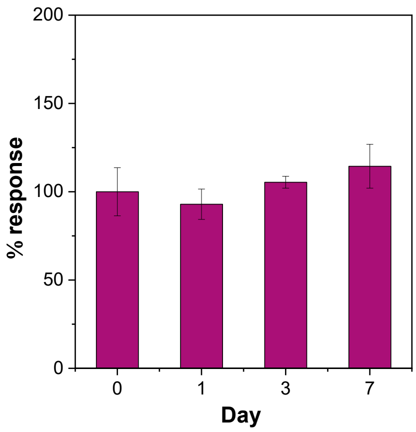

Fig. S5. Stability test for the PQ20-based biosensor against 1:50 diluted serum in PBS throughout 7 days.

Table S2. *Literature available biosensors applied for the seroconversion detection of visceral leishmaniasis from the years 2015-2025.*

Detection method	Platform	Bioreceptor	Sample targeted	LOD (nmol L^{-1})	Ref.
SPR	Au/CYS/PAMAM(G4)	Recombinant protein (C1)	Canine sera	7.37	41
SPR/QCM	Au/MUA	Recombinant chimeric protein (CP10)	Buffer solution	4.23 (SPR) 4.61 (QCM)	30
QCM	Au/Nafion/AuNP-CYS	Recombinant protein (rLci2B)	Canine sera	-	42
PEC	ITO/CdS/AuNP-MPA	Recombinant protein (C8)	Canine sera	0.41	43
PEC	ITO/ZnO/CdS	Synthetic Peptides (PEP13 and PEP16)	Canine sera	-	23
EIS	SPAuE/MPA	Crude soluble antigen (CrudeAg)	Canine sera	-	44
EIS	SPCE	Recombinant Proteins (rLci1A/rLci2B)	Human and Canine sera	-	45
CV	SPCE/AuNPs	Crude soluble antigen (CrudeAg)	Human sera	200 (ng mL^{-1})	46
EIS	SPAuE/Cu-(NH2-BDC)-MOF	KMP-11 antigen	Buffer solution	1:1500	47
SPR	Au/MPA:MUA	Synthetic Peptides (PEP13 and PEP16)	Canine sera	1.05	32
DPV	GCE/$CoFe_2O_4$-C_{60}	Protein A Peptide (Apg)	Human Serum	30.34 (fg mL^{-1})	48
CV	SPCE/GO/AuNPs	Crude soluble antigen (CrudeAg)	Human Serum	5.58 (mg mL^{-1})	49
DPV	GDQ and GO	Peptides (395-G and 395-KKG)	Human and Canine sera	-	50
SPR	Au/MPA:MUA	Recombinant chimeric protein (PQ20)	Canine sera	5.1	This work

Abbreviations: *AuNPs, Gold nanoparticle; CYS, Cysteamine; GCE, Glassy carbon electrode; GO, Graphene oxide; GDQ, graphene quantum dots; ITO, Indium tin oxide; PEC, photoelectrochemical cell; PAMAM(G4), Generation 4 poly(amidoamine) dendrimer; SPAuE, Screen-printed gold electrode; SPCE, Screen-printed carbon electrode.*

SOM approach

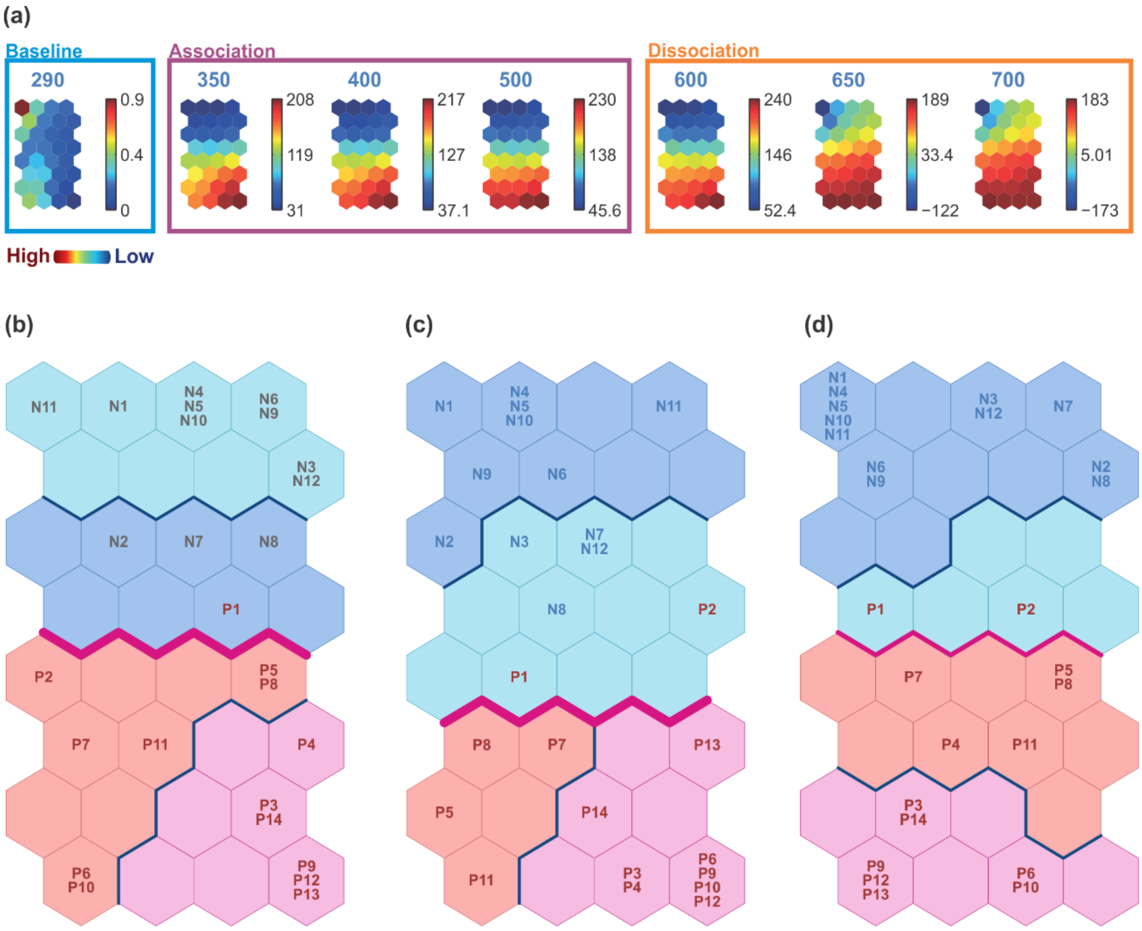

Fig. S6. (a) Component planes representing the θ_{SPR} variation for the analyzed samples in each highlighted time of association time from 290 to 700 s. and SOM grid of the canine serum samples considering **(b)** whole semsorgram, **(c)** dissociation phase (from 590 to 690 s) and **(d)** association interval from 391 s to 490 s.

REFERENCES:

(23) Neto, S. Y.; da Silva, F. G. S.; Souto, D. E. P.; Faria, A. R.; de Andrade, H. M.; de Cássia Silva Luz, R.; Kubota, L. T.; Damos, F. S. Photoelectrochemical immunodiagnosis of canine leishmaniasis using cadmium-sulfide-sensitized zinc oxide modified with synthetic peptides. *Electrochem. Commun.* **2017**, *82* (June), 75–79. https://doi.org/10.1016/j.elecom.2017.07.027.

(30) Souto, D. E. P.; Faria, A. R.; de Andrade, H. M.; Kubota, L. T. Using QCM and SPR for the kinetic evaluation of the binding between a new recombinant chimeric protein and specific antibodies of the visceral leishmaniasis. *Curr. Protein Pept. Sci.* **2015**, *16* (8), 782–790. https://doi.org/10.2174/1389203716666150505230416.

(32) Volpe, J.; Parchen, G. P.; Costa, F. S.; Silva, A. de S.; Andrade, H. M.; Amaral, C. D. B.; Silva, S. M.; Kubota, L. T.; Souto, D. E. P. Synthetic peptides-based SPR biosensor evaluation towards canine visceral leishmaniasis diagnosis: A simple and effective approach. *Microchem. J.* **2024**, *203* (Jan), 110844. https://doi.org/10.1016/j.microc.2024.110844.

(39) Stupin, D. D.; Kuzina, E. A.; Abelit, A. A.; Emelyanov, A. K.; Nikolaev, D. M.; Ryazantsev, M. N.; Koniakhin, S. V.; Dubina, M. V. Bioimpedance spectroscopy: Basics and applications. *ACS Biomater. Sci. Eng.* **2021**, *7* (6). https://doi.org/10.1021/acsbiomaterials.0c01570.

(40) Strong, M. E.; Richards, J. R.; Torres, M.; Beck, C. M.; La Belle, J. T. Faradaic electrochemical impedance spectroscopy for enhanced analyte detection in diagnostics. *Biosens. Bioelectron.* **2021**, *177* (Dec 2020), 112949. https://doi.org/10.1016/j.bios.2020.112949.

(41) Kirchhain, A.; Bonini, A.; Vivaldi, F.; Poma, N.; Di Francesco, F. Latest developments in non-Faradaic impedimetric biosensors: Towards clinical applications. *TrAC, Trends Anal. Chem.* **2020**, *133*, 116073. https://doi.org/10.1016/j.trac.2020.116073.

(42) Ramos-Jesus, J.; Pontes-de-Carvalho, L. C.; Melo, S. M. B.; Alcântara-Neves, N. M.; Dutra, R. F. A gold nanoparticle piezoelectric immunosensor using a recombinant antigen for detecting *Leishmania infantum* antibodies in canine serum. *Biochem. Eng. J.* **2016**, *110*, 43–50. https://doi.org/10.1016/j.bej.2016.01.027.

(43) Neto, S. Y.; Souto, D. E. P.; de Andrade, H. M.; de Cássia Silva Luz, R.; Kubota, L. T.; Damos, F. S. Visible LED light driven photoelectroanalytical detection of antibodies of visceral leishmaniasis based on electrodeposited CdS film sensitized with Au nanoparticles. *Sens. Actuators, B Chem.* **2018**, *256*, 682–690. https://doi.org/10.1016/j.snb.2017.09.202.

(44) Cordeiro, T. A. R.; Gonçalves, M. V. C.; Franco, D. L.; Reis, A. B.; Martins, H. R.; Ferreira, L. F. Label-free electrochemical impedance immunosensor based on modified screen-printed gold electrodes for the diagnosis of canine visceral leishmaniasis. *Talanta* **2019**, *195* (Aug 2018), 327–332. https://doi.org/10.1016/j.talanta.2018.11.087.

(45) Cordeiro, T. A. R.; Martins, H. R.; Franco, D. L.; Santos, F. L. N.; Celedon, P. A. F.; Cantuária, V. L.; de Lana, M.; Reis, A. B.; Ferreira, L. F. Impedimetric immunosensor for rapid and simultaneous detection of Chagas and visceral leishmaniasis for point of care diagnosis. *Biosens. Bioelectron.* **2020**, *169* (Aug), 112573. https://doi.org/10.1016/j.bios.2020.112573.

(46) Martins, B. R.; Barbosa, Y. O.; Andrade, C. M. R.; Pereira, L. Q.; Simão, G. F.; de Oliveira, C. J.; Correia, D.; Oliveira, R. T. S.; da Silva, M. V.; Silva, A. C. A.; Dantas, N. O.; Rodrigues, V.; Muñoz, R. A. A.; Alves-Balvedi, R. P. Development of an electrochemical immunosensor for specific detection of visceral leishmaniasis using gold-modified screen-printed carbon electrodes. *Biosensors* **2020**, *10* (8), 7–8. https://doi.org/10.3390/BIOS10080081.

(47) Perk, B.; Tepeli Büyüksünetçi, Y.; Bachraoui Bouzaien, S.; Diouani, M. F.; Anik, Ü. Fabrication of metal–organic framework based electrochemical *Leishmania* immunosensor. *Microchem. J.* **2023**, *192* (June), 108958. https://doi.org/10.1016/j.microc.2023.108958.

(48) Adu, D. K.; Nate, Z.; Alake, J.; Ike, B. W.; Mahlalela, M. C.; Mohite, S. B.; Mokoena, S.; Chauhan, R.; Karpoormath, R. Rapid and label-free A2 peptide epitope decorated $CoFe_2O_4$-C60 nanocomposite-based electrochemical immunosensor for detecting visceral leishmaniasis. *Bioelectrochemistry* **2024**, *157* (Jan), 108662. https://doi.org/10.1016/j.bioelechem.2024.108662.

(49) Martins, B. R.; Andrade, C. M. R.; Simão, G. F.; Martins, R. de P.; Severino, L. B.; Tanaka, S. C. S. V.; Pereira, L. Q.; da Silva, M. V.; de Vito, F. B.; de Oliveira, C. J. F.; de Souza, H. M.; Lima, A. B.; Júnior, V. R.; Junior, J. R. S.; Alves, R. P. A comparative study of graphene-based electrodes for electrochemical detection of visceral leishmaniasis in symptomatic and asymptomatic patients. *Talanta Open* **2024**, *10* (June). https://doi.org/10.1016/j.talo.2024.100339.

(50) Braz, B. A.; Hospinal-Santiani, M.; Martins, G.; Beirão, B. C. B.; Bergamini, M. F.; Marcolino-Junior, L. H.; Soccol, C. R.; Thomaz-Soccol, V. Disposable electrochemical platform based on solid-binding peptides and carbon nanomaterials: An alternative device for leishmaniasis detection. *Microchim. Acta* **2023**, *190* (8). https://doi.org/10.1007/s00604-023-05891-z.
